# Knowledge mapping of programmed cell death in osteonecrosis of femoral head: a bibliometric analysis (2000–2022)

**DOI:** 10.1186/s13018-023-04314-2

**Published:** 2023-11-14

**Authors:** Xue-Zhen Liang, Nan Li, Jin-Lian Chai, Wei Li, Di Luo, Gang Li

**Affiliations:** 1 First College of Clinical Medicine, Orthopaedic Microsurgery, Affiliated Hospital of Shandong University of Traditional Chinese Medicine, Jingshi Road, 16369, Jinan, 250014 Shandong China; 2 The First Clinical Medical School, Shandong University of Traditional Chinese Medicine, Jinan , 250355 Shandong China; 3 Orthopedics, Liaocheng Hospital of Traditional Chinese Medicine, Liaocheng, 252000 Shandong China; 4 College of Pharmacy, Shandong University of Traditional Chinese Medicine, Jinan, 250355 Shandong China; 5 College of Traditional Chinese Medicine, Shandong University of Traditional Chinese Medicine, Jinan, 250355 Shandong China

**Keywords:** Osteonecrosis of femoral head, Programmed cell death, Apoptosis, Autophagy, CiteSpace, VOSviewers, Bibliometrix

## Abstract

**Background:**

Osteonecrosis of the femoral head (ONFH) is a common, refractory and disabling disease of orthopedic department, which is one of the common causes of hip pain and dysfunction. Recent studies have shown that much progress has been made in the research of programmed cell death (PCD) in ONFH. However, there is no bibliometric analysis in this research field. This study aims to provide a comprehensive overview of the knowledge structure and research hot spots of PCD in ONFH through bibliometrics.

**Method:**

The literature search related to ONFH and PCD was conducted on the Web of Science Core Collection (WoSCC) database from 2002 to 2021. The VOSviewers, “bibliometrix” R package and CiteSpace were used to conduct this bibliometric analysis.

**Results:**

In total, 346 articles from 27 countries led by China and USA and Japan were included. The number of publications related to PCD in ONFH is increasing year by year. Shanghai Jiao Tong University, Xi An Jiao Tong University, Wuhan University and Huazhong University of Science and Technology are the main research institutions. Molecular Medicine Reports is the most popular journal in the field of PCD in ONFH, and Clinical Orthopaedics and Related Research is the most cocited journal. These publications come from 1882 authors among which Peng Hao, Sun Wei, Zhang Chang-Qing, Zhang Jian and Wang Kun-zheng had published the most papers and Ronald S Weinstein was cocited most often. Apoptosis, osteonecrosis, osteonecrosis of the femoral head, glucocorticoid and femoral head appeared are the main topics the field of PCD in ONFH. Autophagy was most likely to be the current research hot spot for PCD in ONFH.

**Conclusion:**

This is the first bibliometric study that comprehensively summarizes the research trends and developments of PCD in ONFH. This information identified recent research frontiers and hot directions, which will provide a reference for scholars studying PCD in ONFH.

## Introduction

Osteonecrosis of the femoral head (ONFH) is a common refractory disease in orthopedics that has an increasing incidence and causes a high disability rate in China [1]. There are numerous treatment options available for ONFH [2], and presently, effective hip preservation therapies consist of core decompression [3] and osteotomy [4]. Nevertheless, the surgical treatment risk escalates with age [5, 6]. ONFH is accompanied by bone marrow composition and bone cell death, which then leads to femoral head structural changes or even femoral head collapse, and cell death marks the initiation and main event of ONFH [2, 7]. Additionally, cell death can occur in a passive manner or through multiple active-mediated cell suicide programs, collectively known as programmed cell death (PCD) [8, 9]. Previous studies have shown that PCD plays an important role in the development of ONFH [10]. In the past two decades, PCD has attracted great attention. In addition to traditional cognitive apoptosis and necrotizing apoptosis, autophagy, pyroptosis, ferroptosis and cuproptosis, which might exist in one or more forms, are interrelated [11, 12]. In-depth investigation of PCD could help to elucidate the molecular mechanism of bone-related cell death in ONFH, reveal its role in the pathogenesis of ONFH, and find new directions and targets for the prevention and treatment of ONFH.

Bibliometrics is based on the characteristics of the literature and adopts statistical analysis methods to quantitatively and qualitatively analyze the distribution structure, quantitative relationship and patterns of change in the literature-related information to provide guidance for future development [13]. When there is a large volume of reference information in the literature, the amount of heavy and complicated information increases the difficulty of the literature analysis. Additionally, bibliometrics can be used to extract literature keywords, sources, authors and publication times, and to perform analyses that reveal the integration and association of information. Furthermore, using visualization methods to more intuitively display analysis results is conducive to the interpretation of a large amount of information and research hot spot mining. Currently, bibliometrics has been applied in fields such as cancer [14], orthopedics [15–18], heart disease [19], nerve disease [20] and autoimmune diseases [21]. Wu et al. summarized the research status of ONFH worldwide, but the study did not introduce the detailed research progress of PCD and ONFH [22]. To our knowledge, no published studies have investigated PCD in ONFH through bibliometric methods. Therefore, to fill this knowledge gap, this study used bibliometric methods and visual analysis to summarize the academic studies of PCD in ONFH over the past two decades (from 2000 to 2022) in the Web of Science database. It aimed to explore the global hot spots and development trends of research and provide relevant guidance for researchers in the field of PCD in ONFH.

## Methods

### Search strategy

**Fig. 1:**
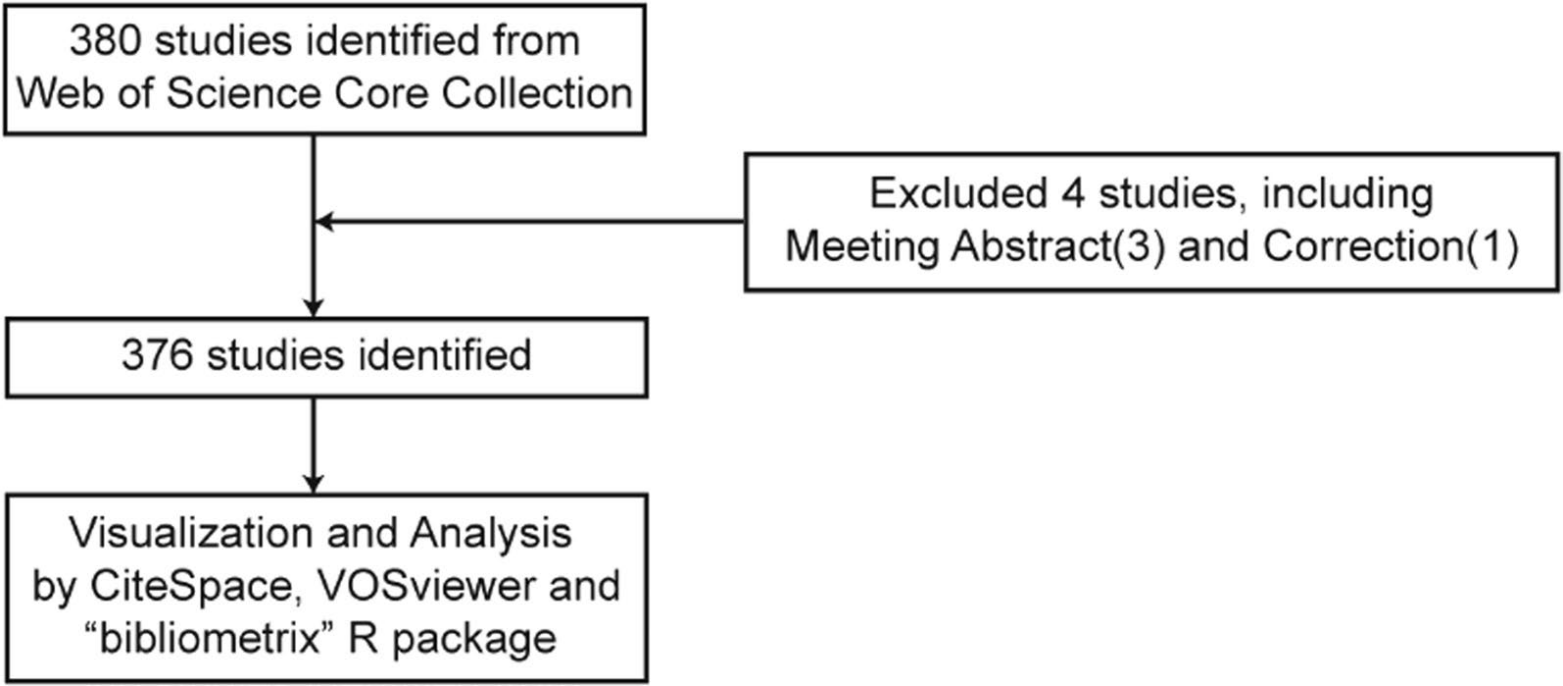
A flow diagrams of publications screening

A literature search related to ONFH and PCD was conducted on the Web of Science Core Collection (WoSCC) database on November 01, 2022 (https://www.webofscience.com/wos/woscc/basic-search). The search formula was ((((((((((((TS = (Femur Head Necrosis)) OR TS = (Femur Head Necroses)) OR TS = (Osteonecrosis of Femoral Head)) OR TS = (Osteonecrosis of Femur Head)) OR TS = (ONFH)) OR TS = (Femoral Head Osteonecrosis)) OR TS = (Femur Head Osteonecrosis)) OR TS = (Femoral Head Necrosis)) OR TS = (Femoral Head Necroses)) OR TS = (Necrosis of Femoral Head)) OR TS = (Necroses of Femoral Head)) OR TS = (Necrosis of Femur Head)) OR TS = (Necroses of Femur Head)) AND ((((TS = (programmed cell death)) OR TS = (apoptosis)) OR TS = (pyroptosis)) OR TS = (autophagy)) OR TS = (ferroptosis)) OR TS = (cuproptosis)), the language was limited to English, the time was set from January 01, 2000, to October 31, 2022, and the type of documents was set to “articles” and “review” (Fig. 1). A complete record of each document was downloaded, including the title, abstract, keywords, year of publication, author, nationality, journal name, research direction, publishing agency, funding agency and references.

### Data analysis

VOSviewer (version 1.6.18, https://www.vosviewer.com/) is a software tool for the construction and visualization of bibliometric networks and provides text mining capabilities that can be used to construct and visualize co-occurrence networks of important terms extracted from the main body of the scientific literature [23–25]. In addition, VOSviewer can process a large amount of information, is easy to understand and can easily provide analysis from multiple angles. In our study, the analyses including country and institution analysis, journal and cocited journal analysis, author and cocited author analysis, and keyword co-occurrence analysis in the ONFH and programmed cell death were completed using VOSviewer based on citations, bibliographic coupling, coreferences or coauthor relationships.

The "bibliometrix" R package (https://www.bibliometrix.org) can be used to perform bibliometric analysis on the relevant literature, which helps us quickly understand the classic literature and the leaders in the field, analyze the future trends, and visualize the results [26]. In our study, the quantitative analysis of publications was conducted with the “bibliometrix” R package (version 3.2.1).

CiteSpace (https://citespace.podia.com/) is a software program developed by Professor Chen Chaomei that can be used to conduct visual analysis of the topics, keywords, journals and other contents of the literature in databases, such as Web of Science, Scopus, PubMed and others [27, 28]. CiteSpace could help researchers quickly clarify the development process of a certain field, find the key literature and major research teams, and identify the research frontiers and development trends in the field; ultimately, it has great potential for improving the efficiency of the literature research. Thus, in our study, the dual-map overlay of journals and references with citation bursts were mainly analyzed by CiteSpace (version 6.1. R4 Basic).

## Results

### Quantitative analysis of publications

**Fig. 2:**
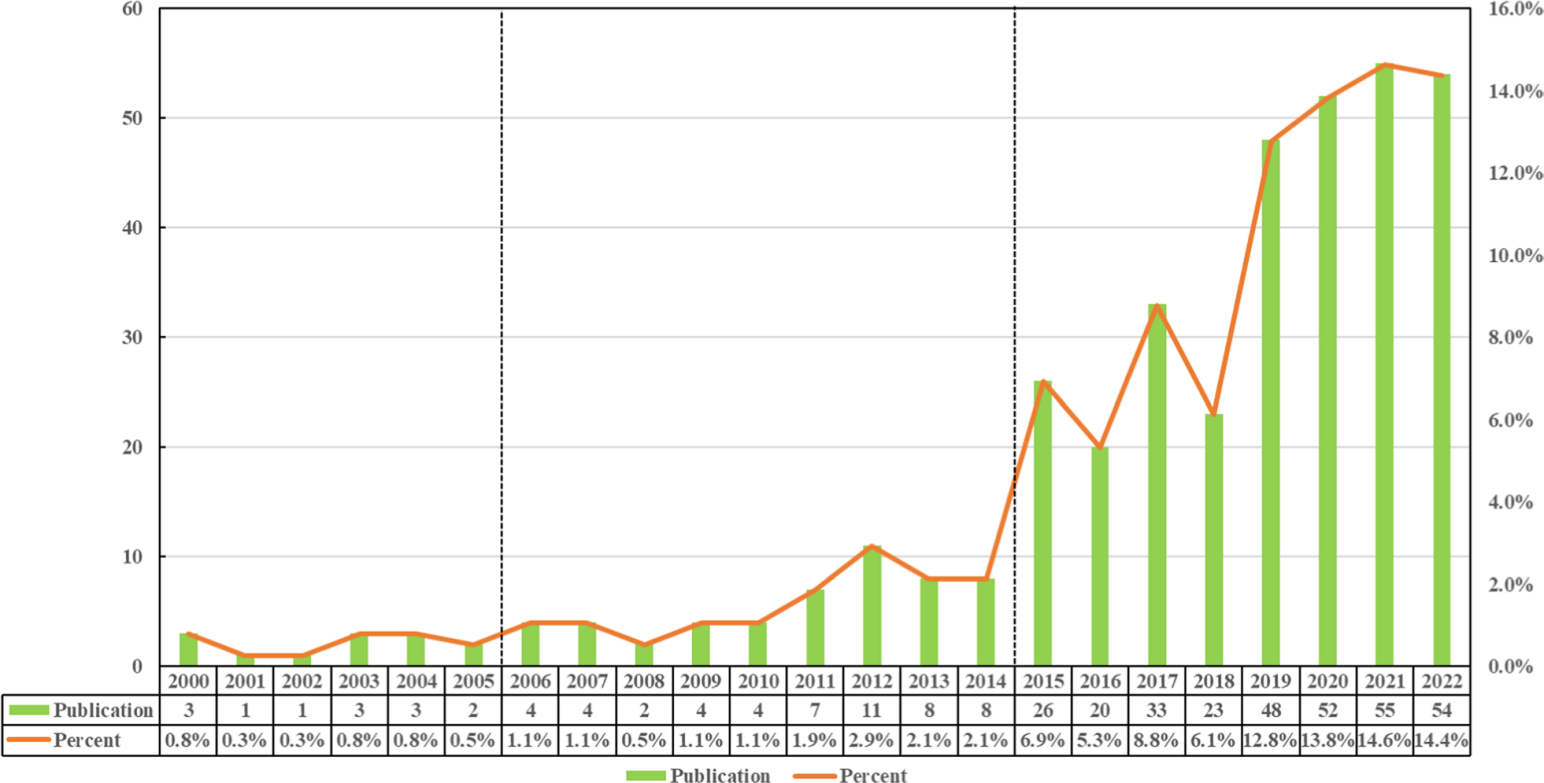
Annual output of research of PCD in ONFH

According to our search results, there were 376 studies of programmed cell death in the past two decades, including 346 “articles” and 30 “reviews.” Judging from the growth rate of the number of publications each year, the whole period can be divided into three parts: Period I (2000–2005), Period II (2006–2014) and III (2015–2022). As shown in Fig. 2, the percentage of papers published in Period I did not exceed 1% per year; during this time, only a few studies on PCD and ONFH were published and were in their infancy. The number of papers published in Period II increased, with an average of between 1 and 5% per year, and it represents the development stage of PCD and ONFH research. The number of publications in Period III began to increase significantly, and it represents the maturation period of PCD and ONFH studies. The number of relevant publications in PCD and ONFH published in 2015 was 26, 3.25 times that of 2014. From January 1, 2022, to October 31, 2022, the number of publications on PCD in ONFH reached 54. In Period III (except for 2016 and 2018), the number of publications on PCD in ONFH increased year by year, and the total number of papers in this stage increased significantly compared with the previous two stages.

### Country and institutional analysis

**Fig. 3:**
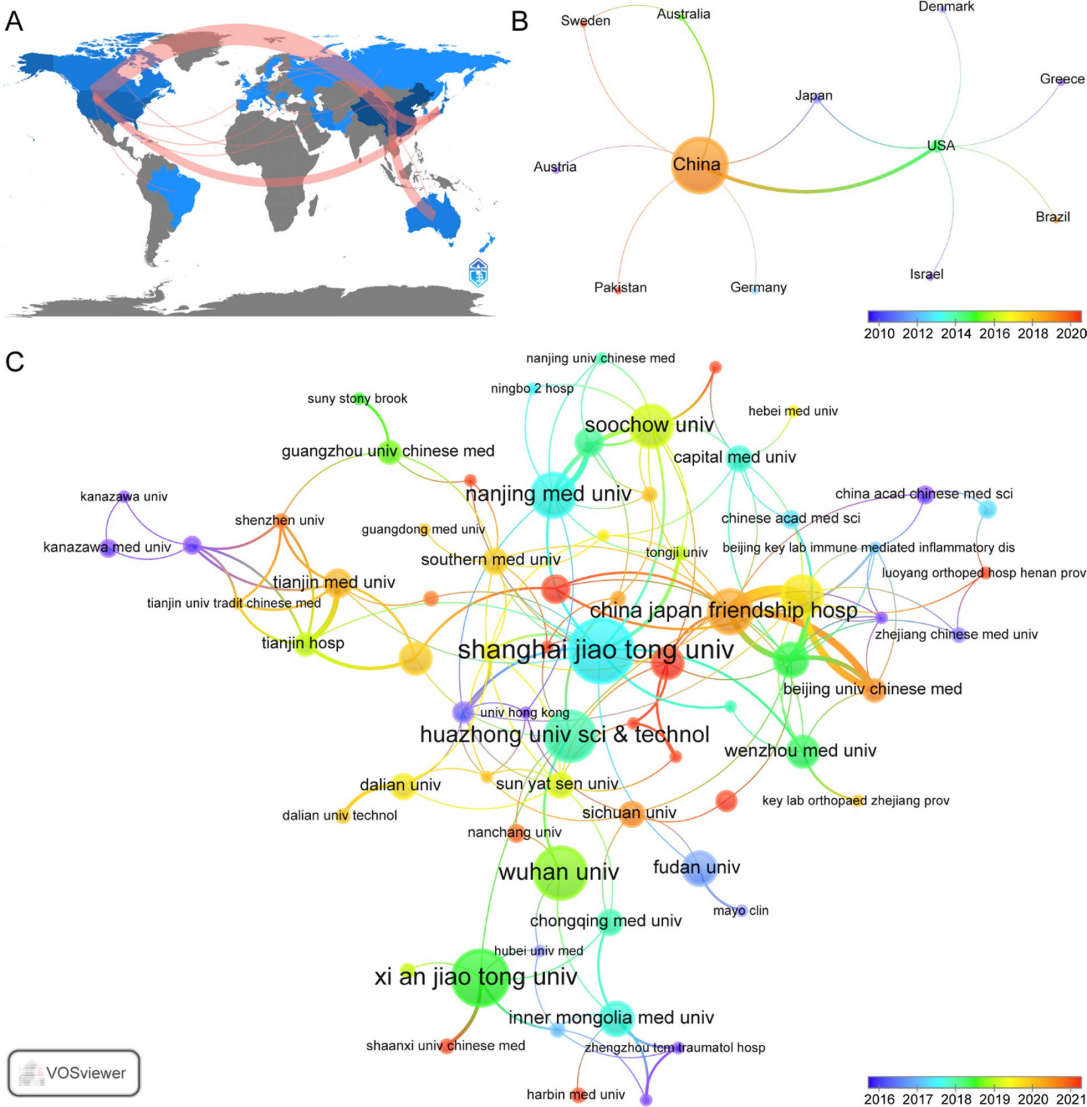
Geographical distribution (**A**) and visualization of countries (**B**) and organizations (**C**) on research of PCD in ONFH. Note: The color of the circles represent different clusters, the number of circles represents the number of countries/organizations analyzed, and the size of the circles represents the proportion of the country/organizations in the analysis: The larger the circle, the greater the contribution. The connections between the circles represent the connections between countries/organizations, and the more or thicker the connections, the closer the connections between the two

These publications were from 27 countries and 355 institutions. The top ten countries were distributed among Asia, Europe, North America and Oceania, and they were mainly in Asia (n = 4) and Europe (n = 3) (Table 1). Among the countries, the one with the largest number of publications was China (n = 307, 76.6%), followed by the USA (n = 30, 7.5%), Japan (n = 17, 4.2%), Belgium (n = 7, 17%) and Canada (n = 5, 1.2%). The combined number of publications from China and the USA accounted for almost half of the total (84%). Subsequently, we filtered and visualized 13 countries that had a number of publications greater than or equal to 2 and constructed a collaborative network based on the number and relationship of publications in each country (Fig. 3B). Notably, there is much active cooperation between different countries. For example, China has close cooperation with the USA (8), Australia (3), Japan (2), Austria (1), Germany (1), Pakistan (1) and Sweden (1); the Italy has active cooperation with Russia (1); Canada has active cooperation with Iran (1); and Australia has active cooperation with Sweden (1).

**Table 1:** Top 10 countries and institutions on research of PCD in ONFH

Rank	Country	Counts	Percent	Institution	Country	Counts (%)
1	China (Asia)	307	76.6	Shanghai jiao Tong univ	26	3.5
2	USA (North America)	30	7.5	Xi an jiao Tong univ	22	3.0
3	Japan (Asia)	17	4.2	Wuhan univ	20	2.7
4	Belgium (Europe)	7	1.7	Huazhong univ sci & technol	19	2.6
5	Canada (North America)	5	1.2	China Japan friendship hosp	16	2.2
6	Australia (Oceania)	4	1.0	Nanjing med univ	16	2.2
7	Iran (Asia)	3	0.7	Soochow univ	15	2.0
8	Italy (Europe)	3	0.7	Peking univ	14	1.9
9	South Korea (Asia)	3	0.7	Fudan univ	11	1.5
10	Austria (Europe)	2	0.5	Inner mongolia med univ	11	1.5

The top 10 institutions are all located in China. The four institutions that published the most relevant papers are Shanghai Jiao Tong University (n = 26, 3.5%), Xi An Jiao Tong University (n = 22, 3.0%), Wuhan University (n = 20, 2.7%) and Huazhong University of Science and Technology (n = 19,2.6%). Subsequently, we selected 90 institutions that had a minimum number of publications equal to 2 for visualization and constructed a collaborative network based on the number and relationship of publications of each institution (Fig. 3C). As shown in Fig. 3C, the cooperation between China-Japan Friendship Hospital, Peking University, Peking Union Medical College and Beijing University of Chinese Medicine is very close. In addition, we note that Shanghai Jiao Tong University has published the largest number of papers and has close partnerships with other institutions, such as Fudan University, Chinese Academy of Sciences, Chinese University of Hong Kong, Wenzhou Medical University and Nanjing Medical University.

### Journals and cocited journals

**Fig. 4:**
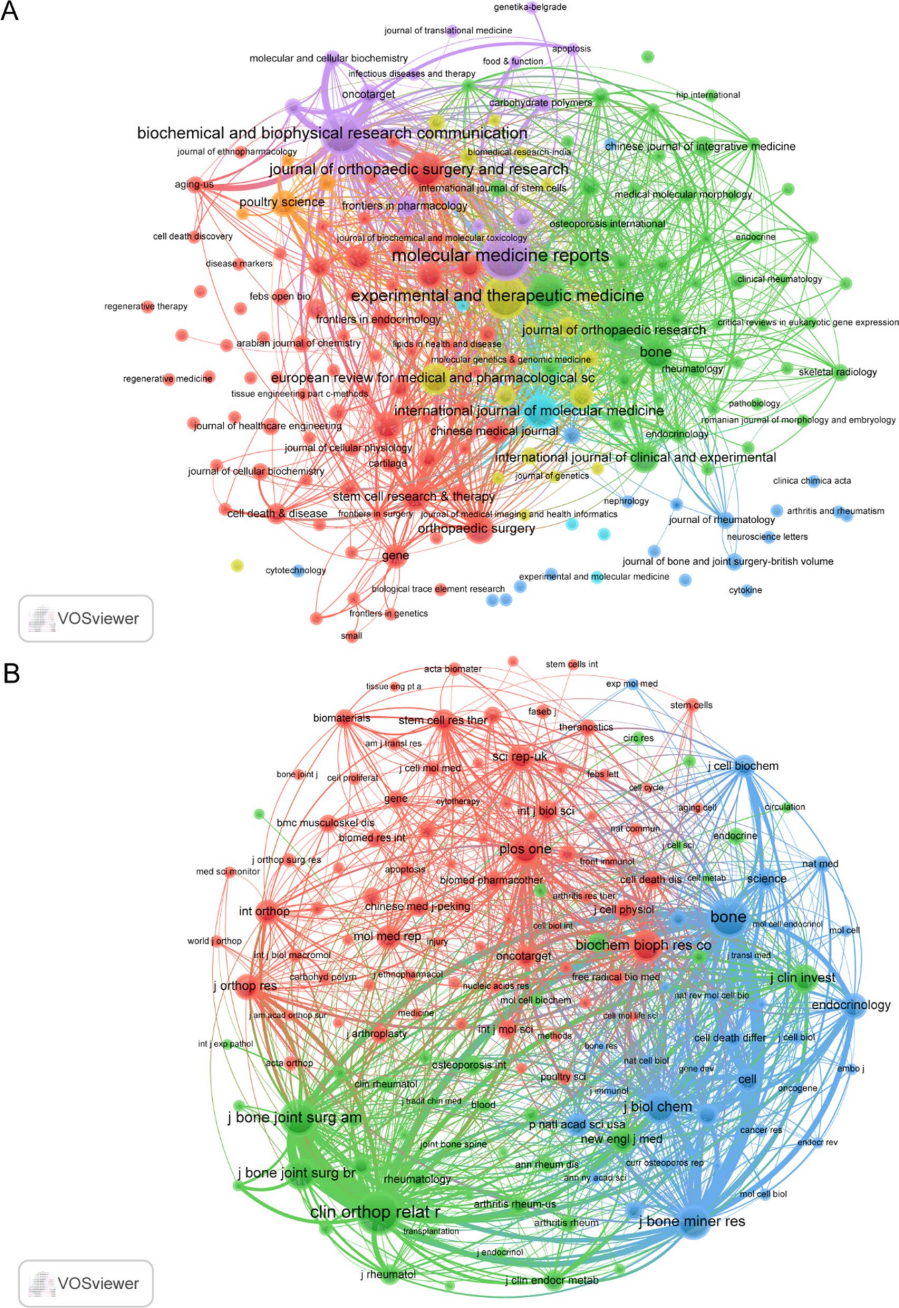
Visualization of journals (**A**) and cocited journals (**B**) on research of PCD in ONFH. Note: The color of the circles represents different clusters, the number of circles represents the number of the analyzed journals, and the size of the circles represents the number of articles published in each journal

**Fig. 5:**
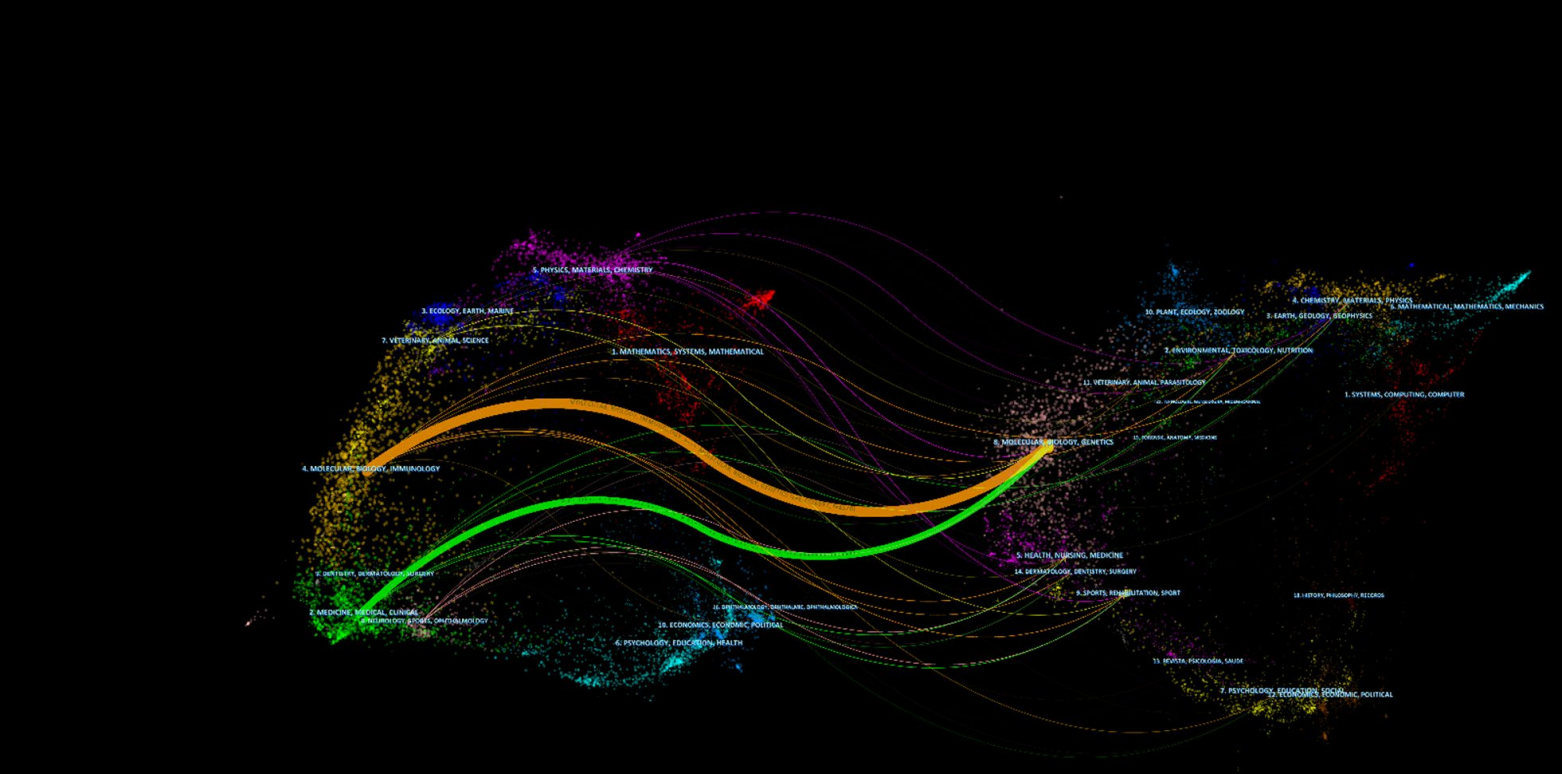
Dual-map overlay of journals on research of PCD in ONFH

Publications related to PCD in ONFH were published in 191 journals. Molecular Medicine Reports published the most papers (n = 15, 4.0%), followed by Experimental and Therapeutic Medicine (n = 13, 3.5%), Biochemical and Biophysical Research Communications (n = 12, 3.2%), BMC Musculoskeletal Disorders (n = 9, 2.4%) and Journal of Orthopaedic Surgery and Research (n = 9, 2.4%). Among the top 20 journals, there were 4 in Q1 and 8 in Q2 in Journal Citation Reporting (JCR), and the journal with the highest impact factor was International Journal of Biological Sciences (IF = 10.75), followed by Stem Cell Research & Therapy (IF = 8.079) and Chinese Medical Journal (IF = 6.133). Subsequently, we screened all journals based on the minimum number of relevant publications equal to 1 and mapped the journal network (Fig. 4A). Figure 5A shows that Molecular Medicine Reports has active citation relationships with Bone, Biochemical and Biophysical Research Communications, Orthopaedic Surgery and Experimental and Therapeutic Medicine, and others.

As shown in Table 2, among the top 20 cocited journals, 3 journals were cited more than 300 times, and Clinical Orthopaedics and Related Research (cocitations = 438) was the most cited journal, followed by Bone (cocitations = 373) and Journal of Bone and Joint Surgery-American Volume (cocitations = 313). In addition, the impact factor of the New England Journal of Medicine is the highest (IF = 176.079), followed by Nature (IF = 69.504) and Cell (IF = 66.85). Journals with a minimum cocitation equal to 20 were filtered to map the cocitation network (Fig. 4B). As shown in Fig. 4B, Bone has positive cocitation relationships with Clinical Orthopaedics and Related Research, Journal of Bone and Joint Surgery-American Volume, Journal of Bone and Mineral Research, and Journal of Orthopaedic Research.

**Table 2:** Top 20 journals and cocited journals for research of PCD in ONFH

Rank	Journal	Count	IF	JCR	Journal	Cocitation	IF	JCR
1	Molecular Medicine Reports	15 (4.0%)	3.423	Q3	Clinical Orthopaedics and Related Research	438 (4.4%)	4.755	Q1
2	Experimental and Therapeutic Medicine	13 (3.5%)	2.751	Q4	Bone	373 (3.7%)	4.626	Q2
3	Biochemical and Biophysical Research Communications	12 (3.2%)	3.322	Q3	Journal of Bone and Joint Surgery-American Volume	313 (3.1%)	6.558	Q1
4	Bmc Musculoskeletal Disorders	9 (2.4%)	2.562	Q3/Q4	Journal of Bone and Mineral Research	283 (2.8%)	6.39	Q1
5	Journal of Orthopaedic Surgery and Research	9 (2.4%)	2.677	Q2	Journal of Biological Chemistry	251 (2.5%)	5.486	Q2
6	Bone	8 (2.1%)	4.626	Q2	Biochemical and Biophysical Research Communications	243 (2.4%)	3.322	Q3
7	International Journal of Molecular Medicine	8 (2.1%)	5.314	Q2	PLoS One	223 (2.2%)	3.752	Q2
8	European Review for Medical and Pharmacological Sciences	7 (1.9%)	3.784	Q2	Journal of Clinical Investigation	183 (1.8%)	19.456	Q1
9	Biomed Research International	6 (1.6%)	3.246	Q3	Endocrinology	160 (1.6%)	5.051	Q2
10	International Journal of Clinical and Experimental Pathology	6 (1.6%)	N/A	Q4	Journal of Orthopaedic Research	156 (1.6%)	3.102	Q2
11	Journal of Orthopaedic Research	6 (1.6%)	3.102	Q2	Scientific reports	156 (1.6%)	4.996	Q2
12	Orthopaedic Surgery	6 (1.6%)	2.279	Q3	New England Journal of Medicine	153 (1.5%)	176.079	Q1
13	International Journal of Clinical and Experimental Medicine	5 (1.3%)	N/A	Q4	Molecular Medicine Reports	144 (1.4%)	3.423	Q3
14	Journal of Cellular and Molecular Medicine	5 (1.3%)	5.295	Q2	International Orthopaedics	141 (1.4%)	3.479	Q2
15	Poultry Science	5 (1.3%)	4.014	Q1	Cell	133 (1.3%)	66.85	Q1
16	Stem Cell Research & Therapy	5 (1.3%)	8.079	Q1	Proceedings of the National Academy of Sciences of the United States of America	124 (1.2%)	12.779	Q1
17	Chinese Medical Journal	4 (1.1%)	6.133	Q1	International Journal of Biological Sciences	123 (1.2%)	10.75	Q1
18	Gene	4 (1.1%)	3.913	Q2	Nature	123 (1.2%)	69.504	Q1
19	International Journal of Biological Sciences	4 (1.1%)	10.75	Q1	Stem cell research & therapy	121 (1.2%)	8.079	Q1
20	Scientific Reports	4 (1.1%)	4.996	Q2	Journal of Cellular Biochemistry	118 (1.2%)	4.48	Q2/Q3

**Fig. 6:**
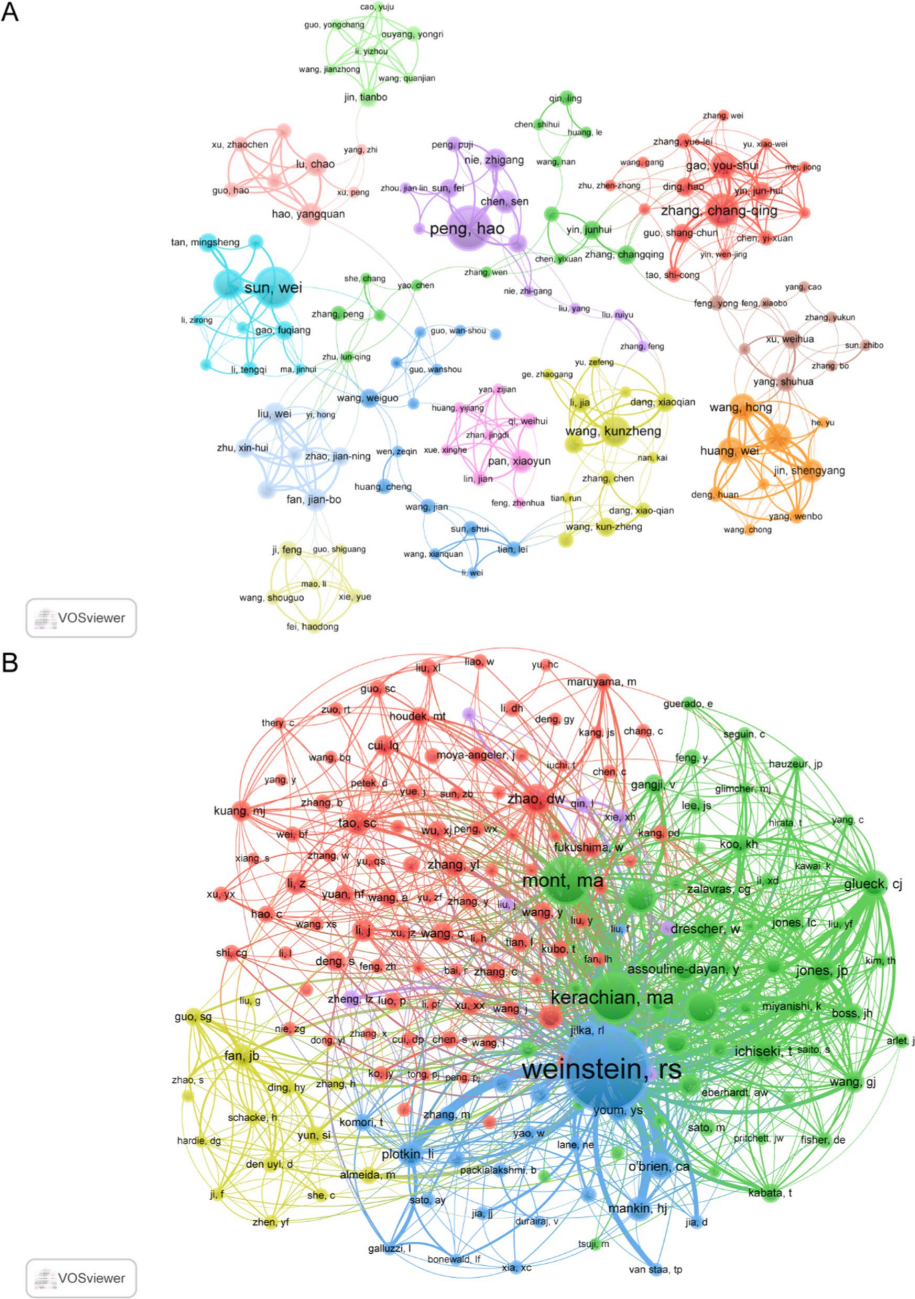
Visualization of authors (**A**) and cocited authors (**B**) on research of PCD in ONFH. Note: The color of circles represents different clusters, and the number of circles represents the number of the author. The size of the analysis of the circle represents the proportion of the author in the analysis. The larger the circle, the greater the contribution. Wires between circles represent the connections between authors, and more or thicker connections represent the closer connections between the two

The dual-map overlay of journals shows the citation relationships between journals and cocited journals, with clusters of citing journals on the left and clusters of cited journals on the right. As shown in Fig. 6, the orange path is the main citation path, which represents the research published in 4 molecular, biology, immunology journals is mainly cited by the literature in 8 molecular, biology, genetics journals.

### Authors and cocited authors

A total of 1882 authors participated in PCD research in ONFH. Among the top 20 authors, each author published no less than 5 papers (Table 3). We built a collaborative network based on authors whose number of published papers was greater than or equal to 2 (Fig. 6A). Peng Hao, Sun Wei, Zhang Chang-Qing, Zhang Jian and Wang Kun-zheng had larger nodes because they had more related publications. In addition, we observed close collaboration among multiple authors. For example, Sun Wei had close cooperation with Wu Xin-jie, Tan Ming-sheng and Hao Yang-quan.

**Table 3:** Top 20 authors and cocited authors on research of PCD in ONFH

Rank	Author	Count	Cocited authors	Citations
1	Peng, Hao	11	Weinstein, Rs	298
2	Sun, Wei	10	Kerachian, Ma	128
3	Zhang, Chang-qing	8	Mont, Ma	123
4	Zhang, Jian	8	Calder, Jdf	67
5	Wang, Kunzheng	7	Hernigou, P	61
6	Wu, Xinjie	7	Zhao, Dw	52
7	Gao, You-shui	6	Ichiseki, T	46
8	Huang, Wei	6	Glueck, Cj	45
9	Meng, Chunqing	6	Jones, Jp	45
10	Wang, Hong	6	Assouline-dayan, Y	43
11	Zhou, Zhenlei	6	Drescher, W	41
12	Chen, Sen	5	o'brien, Ca	41
13	Fan, Jian-bo	5	Plotkin, Li	40
14	Gangji, Valerie	5	Yamamoto, T	4
15	Hao, Yangquan	5	Mankin, Hj	37
16	Harvey, Edward j	5	Okazaki, S	37
17	Jin, Shengyang	5	Tao, Sc	36
18	Kerachian, Mohammad amin	5	Fan, Jb	34
19	Li, Tao	5	Zhang, Yl	34
20	Liu, Wei	5	Youm, Ys	33

Among the 8495 cocited authors, 14 authors were cocited more than 40 times (Table 3). The most frequently cocited author is Ronald S Weinstein (n = 295), followed by Mohammad Amin Kerachian (n = 128) and Michael A Mont (n = 123). Authors with minimum cocitations equal to 10 were filtered to map cocitation network graphs (Fig. 6B). As shown in Fig. 6B, there are also active collaborations among different cocited authors, such as Ronald S Weinstein and Michael A Mont and Mohammad Amin Kerachian.

### Cited references

**Fig. 7:**
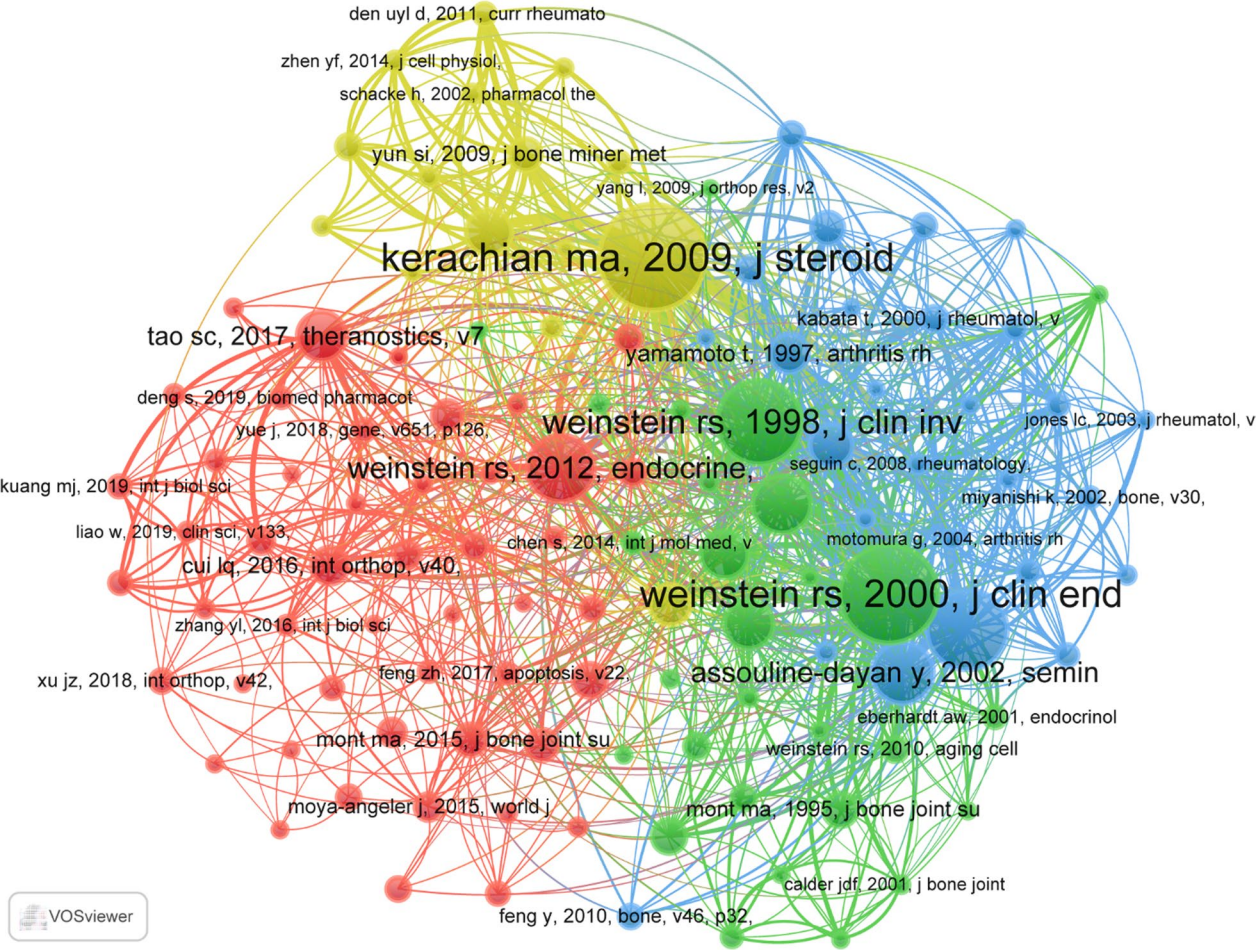
Visualization of cocited references on research of PCD in ONFH. Note: The color of the circles represents different clusters, the number of the circles represents the number of the analyzed cocited references, and the size of the circles represents the number of citations of each cocited reference. The interconnected two points represent two documents simultaneously cited by another paper. The length of the connection represents the correlation of the two cocited references, and the stronger the correlation, the shorter the connection

**Fig. 8:**
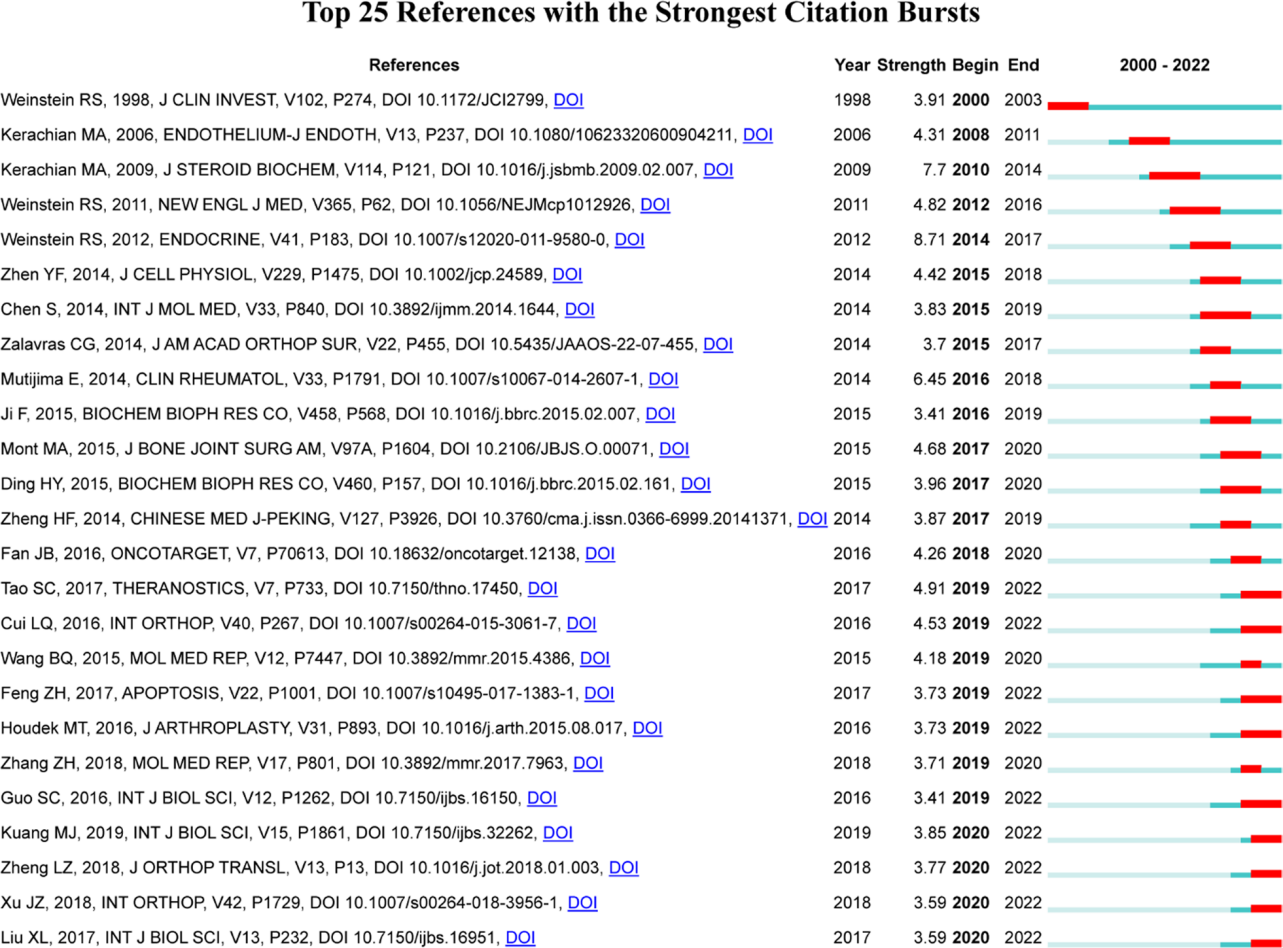
Top 25 references with strong citation bursts of PCD in ONFH. A red bar indicates high citations in that year

There have been 10,734 cocited references on PCD research in ONFH over the past two decades. In the top 20 cocited references (Table 4), all references were cocited at least 23 times, and four references were cocited more than 50 times. We selected references with cocitations greater than or equal to 10 for the construction of the cocitation network map (Fig. 7). According to Fig. 8, “Weinstein Rs, 2000, J Clin Endocr Metab” shows active cocited relationships with “Kerachian Ma, 2009, J Steroid Biochem,” “Weinstein Rs, 1998, J Clin Invest” and “Tao Sc, 2017, Theranostics.”

**Table 4:** Top 20 cocited references on research of PCD in ONFH

Rank	Cited reference	Citations
1	Kerachian ma, 2009, j steroid biochem, v114, p121, https://doi.org/10.1016/j.jsbmb.2009.02.007[29]	74
2	Weinstein rs, 2000, j clin endocr metab, v85, p2907, https://doi.org/10.1210/jc.85.8.2907[30]	69
3	Weinstein rs, 1998, j clin invest, v102, p274, https://doi.org/10.1172/jci2799[31]	59
4	Calder jdf, 2004, j bone joint surg br, v86b, p1209, https://doi.org/10.1302/0301-620x.86b8.14834[32]	56
5	Weinstein rs, 2012, endocrine, v41, p183, https://doi.org/10.1007/s12020-011-9580-0[33]	47
6	Assouline-dayan y, 2002, semin arthritis rheu, v32, p94, https://doi.org/10.1053/sarh.2002.33724[34]	43
7	o'brien ca, 2004, endocrinology, v145, p1835, https://doi.org/10.1210/en.2003-0990[35]	41
8	Mankin hj, 1992, new engl j med, v326, p1473 [36]	34
9	Tao sc, 2017, theranostics, v7, p733, https://doi.org/10.7150/thno.17450[37]	34
10	Youm ys, 2010, clin orthop surg, v2, p250, https://doi.org/10.4055/cios.2010.2.4.250[38]	33
11	Weinstein rs, 2011, new engl j med, v365, p62, https://doi.org/10.1056/nejmcp1012926[39]	32
12	Kerachian ma, 2006, endothelium-j endoth, v13, p237, https://doi.org/10.1080/10623320600904211[40]	30
13	Mont ma, 2006, j bone joint surg am, v88a, p1117, https://doi.org/10.2106/jbjs.e.01041[41]	29
14	Yamamoto t, 1997, arthritis rheum, v40, p2055, https://doi.org/10.1002/art.1780401119[42]	27
15	Cui lq, 2016, int orthop, v40, p267, https://doi.org/10.1007/s00264-015-3061-7[43]	26
16	Livak kj, 2001, methods, v25, p402, https://doi.org/10.1006/meth.2001.1262[44]	24
17	Mont ma, 1995, j bone joint surg am, v77a, p459, https://doi.org/10.2106/00004623-199503000-00018[45]	24
18	Mutijima e, 2014, clin rheumatol, v33, p1791, https://doi.org/10.1007/s10067-014-2607-1[46]	24
19	Mont ma, 2015, j bone joint surg am, v97a, p1604, https://doi.org/10.2106/jbjs.o.00071[47]	23
20	Yun si, 2009, j bone miner metab, v27, p140, https://doi.org/10.1007/s00774-008-0019-5[48]	23

## References with citation bursts

References with citation bursts refer to those references that are frequently cited by scholars in a certain field over an interval of time. In our study, 25 references with strong citation bursts were identified by CiteSpace (Fig. 8 and Table 5). As shown in Fig. 8, every bar represents a year, and the red bar represents strong citation burstness. Citation bursts for references appeared as early as 2000 and as late as 2020. The reference with the strongest citation burst (strength = 8.71) was titled “Glucocorticoid-induced osteonecrosis,” authored by Robert S. Weinstein, with citation bursts from 2014 to 2017. The reference with the second strongest citation burst (strength = 7.7) was titled “Glucocorticoids in osteonecrosis of the femoral head: A new understanding of the mechanisms of action,” authored by Mohammad Amin Kerachian et al., with citation bursts from 2010 to 2014. Overall, the burst strength of these 13 references ranged from 3.41 to 8.71, and the endurance strength ranged from 1 to 4 years.

**Table 5:** Title of the 25 references with strong citations bursts of PCD in ONFH

Rank	Strength	Title
1	3.91	Inhibition of osteoblastogenesis and promotion of apoptosis of osteoblasts and osteocytes by glucocorticoids. Potential mechanisms of their deleterious effects on bone [31]
2	4.31	Avascular necrosis of the femoral head: vascular hypotheses [40]
3	7.7	Glucocorticoids in osteonecrosis of the femoral head: A new understanding of the mechanisms of action [29]
4	4.82	Clinical practice. Glucocorticoid-induced bone disease [39]
5	8.71	Glucocorticoid-induced osteonecrosis [33]
6	4.42	P53 dependent mitochondrial permeability transition pore opening is required for dexamethasone-induced death of osteoblasts [49]
7	3.83	Administration of erythropoietin exerts protective effects against glucocorticoid-induced osteonecrosis of the femoral head in rats [50]
8	3.7	Osteonecrosis of the femoral head: evaluation and treatment [51]
9	6.45	The apoptosis of osteoblasts and osteocytes in femoral head osteonecrosis: its specificity and its distribution [46]
10	3.41	K6PC-5, a novel sphingosine kinase 1 (SphK1) activator, alleviates dexamethasone-induced damages to osteoblasts through activating SphK1-Akt signaling [52]
11	4.68	Nontraumatic Osteonecrosis of the Femoral Head: Where Do We Stand Today? A Ten-Year Update [47]
12	3.96	Dexamethasone-induced apoptosis of osteocytic and osteoblastic cells is mediated by TAK1 activation [53]
13	3.87	Gastrodin prevents steroid-induced osteonecrosis of the femoral head in rats by anti-apoptosis [54]
14	4.26	miR-135b expression downregulates Ppm1e to activate AMPK signaling and protect osteoblastic cells from dexamethasone [55]
15	4.91	Exosomes derived from human platelet-rich plasma prevent apoptosis induced by glucocorticoid-associated endoplasmic reticulum stress in rat osteonecrosis of the femoral head via the Akt/Bad/Bcl-2 signal pathway [37]
16	4.53	Multicentric epidemiologic study on six thousand three hundred and ninety five cases of femoral head osteonecrosis in China [43]
17	4.18	MicroRNA expression in bone marrow mesenchymal stem cells from mice with steroid-induced osteonecrosis of the femoral head [56]
18	3.73	Fludarabine inhibits STAT1-mediated up-regulation of caspase-3 expression in dexamethasone-induced osteoblasts apoptosis and slows the progression of steroid-induced avascular necrosis of the femoral head in rats [57]
19	3.73	Decreased Osteogenic Activity of Mesenchymal Stem Cells in Patients With Corticosteroid-Induced Osteonecrosis of the Femoral Head [58]
20	3.71	MicroRNA-206 contributes to the progression of steroid-induced avascular necrosis of the femoral head by inducing osteoblast apoptosis by suppressing programmed cell death 4 [59]
21	3.41	Exosomes from Human Synovial-Derived Mesenchymal Stem Cells Prevent Glucocorticoid-Induced Osteonecrosis of the Femoral Head in the Rat [60]
22	3.85	Exosomes derived from Wharton's jelly of human umbilical cord mesenchymal stem cells reduce osteocyte apoptosis in glucocorticoid-induced osteonecrosis of the femoral head in rats via the miR-21-PTEN-AKT signalling pathway [61]
23	3.77	Steroid-associated osteonecrosis animal model in rats [62]
24	3.59	Animal models of steroid-induced osteonecrosis of the femoral head-a comprehensive research review up to 2018 [63]
25	3.59	Exosomes Secreted from Human-Induced Pluripotent Stem Cell-Derived Mesenchymal Stem Cells Prevent Osteonecrosis of the Femoral Head by Promoting Angiogenesis [64]

### Hot spots and frontiers

Through the co-occurrence analysis of keywords, we could quickly capture research hot spots in a certain field. There are 790 author keywords on research on PCD in ONFH over the past two decades. Table 6 shows the top 40 high-frequency keywords in research on PCD in ONFH. Among these keywords, apoptosis, osteonecrosis, osteonecrosis of the femoral head, glucocorticoid and femoral head appeared more than 30 times, and they represented the main research directions of PCD in ONFH.

**Table 6:** Top 40 keywords on research of PCD in ONFH

Rank	Keywords	Counts	Rank	Keywords	Counts
1	Apoptosis	94	21	Hypoxia	9
2	Osteonecrosis	71	22	Mesenchymal stem cells	9
3	Osteonecrosis of the femoral head	44	23	Osteonecrosis of femoral head	9
4	Glucocorticoid	39	24	Bone marrow mesenchymal stem cells	8
5	Femoral head	30	25	Osteogenic differentiation	8
6	Dexamethasone	27	26	Broiler	7
7	Osteoblast	26	27	PI3K	7
8	Osteoblasts	22	28	Bone microvascular endothelial cells	6
9	Autophagy	18	29	Microrna	6
10	Femoral head necrosis	18	30	mTOR	6
11	Angiogenesis	14	31	Necrosis	6
12	Steroid-induced osteonecrosis of the femoral head	14	32	Osteogenesis	6
13	Glucocorticoids	13	33	Proliferation	6
14	Steroid	13	34	Rat	6
15	Akt	11	35	SONFH	6
16	Oxidative stress	11	36	Steroid-induced avascular necrosis of the femoral head (SANFH)	6
17	Exosomes	10	37	Avascular necrosis of the femoral head	5
18	ONFH	10	38	caspase-3	5
19	Steroid-induced avascular necrosis of the femoral head	10	39	Extracellular vesicles	5
20	Avascular necrosis	9	40	HIF-1 alpha	5

**Fig. 9:**
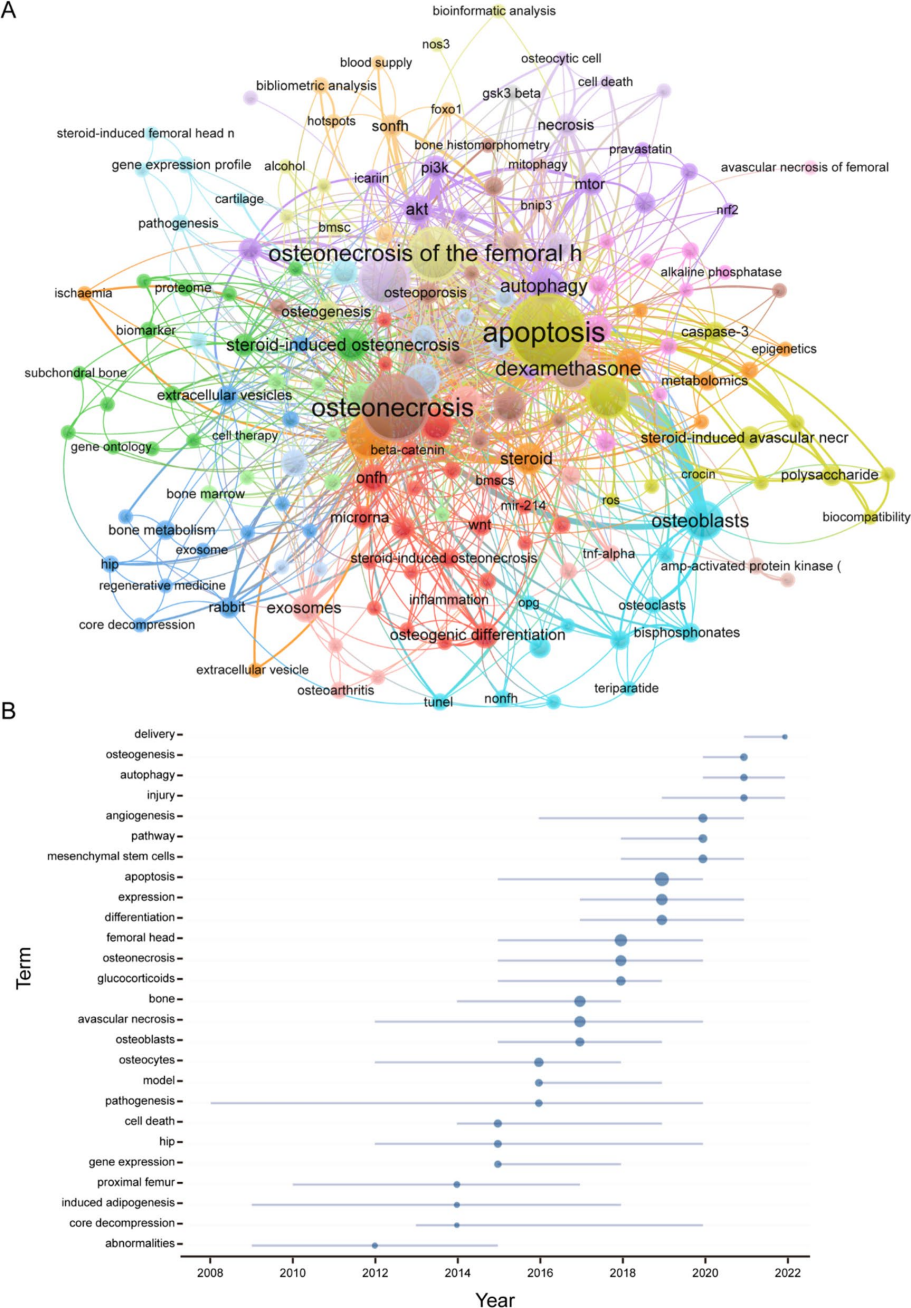
Keyword cluster analysis (**A**) and trend topic analysis (**B**) of PCD in ONFH

We filtered keywords with numerous occurrences greater than or equal to 2 and performed cluster analysis through VOSviewer (Fig. 9A). As shown in Fig. 9A, we obtained 19 clusters in total, representing 19 research directions. The keywords in the yellow clusters consist of apoptosis, biocompatibility, Caspase-3, crocin, femoral necrosis, Jnk/c-jun signaling pathway, etc. The keywords in the red clusters consist of angiogenesis, beta-catenin, BMSCs, cell proliferation, circular RNA, microRNA, etc. The keywords in the green clusters consist of alendronate, articular cartilage, biomarker, bone, differentially expressed genes, rat, serum, gene ontology, systematic review, Traditional Chinese Medicine, etc. The keywords in the blue clusters consist of animal model, bone loss, bone metabolism, core decompression, exosome, rabbit, tissue engineering, etc.

The analysis of the topic trends of the keywords (Fig. 9B) showed that from 2009 to 2015, the research in this period mainly focused on abnormalities, core decompression and gene expression and that the main keywords were abnormalities, proximal femur, induced adipogenesis, hip, core decompression and gene expression. Since 2016, scholars have been actively exploring the pathogenesis and model of PCD in ONFH, and the main keywords are pathogenesis, osteocytes, model, etc.

## Discussion

In the past two decades, research on PCD in ONFH has increased in depth and breadth, and it has aligned with current development trends and provided new ideas and methods for the diagnosis and treatment of ONFH. Moreover, this paper analyzes the research hot spots, frontiers and trends of PCD in ONFH.

There were no more than three annual publications on this topic from 2000–2005, indicating that there were few studies on PCD in ONFH and that the foundation of research between ONFH and PCD was in its infancy during this period. From 2006 to 2014, there were still an average of 5.7 papers published in this field per year. From 2015 to 2022, the number of publications began to increase substantially, with an average of 38.9 papers being published per year. In the past eight years, the number of related documents has grown rapidly, indicating that the study of PCD in ONFH is in a mature period and that the related research has attracted increasing attention from scholars.

China, the USA and Japan are the major countries conducting studies of PCD in ONFH. In terms of the quantity and quality of the literature published, China ranks first in the world and is far ahead of the USA, which ranks at no. 2; this is evidence that China has led the world in the field of ONFH in the past five years, which may be closely related to the large number of patients with ONFH in China. We have noted the close cooperation between China, the USA and Japan. In addition, Australia has actively cooperated with China and Sweden. In research institutions, all of the top 10 research institutions were located in China, and there was good cooperation among some of them, such as China-Japan Friendship Hospital, Peking University, Peking Union Medical College and Beijing University of Chinese Medicine. In addition, we also found that most papers were published by Shanghai Jiao Tong University. At the same time, there was also more cooperation with other institutions, such as Fudan University, the Chinese Academy of Sciences, the Chinese University of Hong Kong, Wenzhou Medical University and Nanjing Medical University. Furthermore, this cooperation was very beneficial for the long-term development of academic research. Although there was cooperation between some countries, for example, there was only a small amount of cooperation between institutions in the USA, China and Japan, and the breadth and intensity of cooperation between these institutions are not ideal. Obviously, in the long run, this situation will not foster development and progress in the research field. Therefore, we strongly recommend that the research institutions of various countries actively conduct extensive cooperation and exchanges to jointly promote in-depth research on PCD in ONFH.

Most studies on PCD in ONFH were published in Molecular Medicine Reports (IF = 3.423, Q3), indicating that it is currently the most popular journal in the research field. Among these journals, the journal with the highest impact factor was International Journal of Biological Sciences (IF = 10.75), followed by Stem Cell Research & Therapy (IF = 8.079) and Chinese Medical Journal (IF = 6.133). For the cocited journals, we found that the majority are high-impact Q1 and Q2 journals in JCR. Clearly, these journals were high-quality international journals, providing support for the study of PCD in ONFH. In addition, the current studies on PCD in ONFH were mainly published in molecular, biology and immunology journals, and the clinically relevant studies were published in very few journals, indicating that most of the research is still in the basic stage of development.

From the author's perspective, Peng Hao, Sun Wei, Zhang Chang-Qing and Zhang Jian had the most published papers, with more than 8 papers per person. For the case of the authors, the most cocited author was Ronald S Weinstein (n = 295), followed by Mohammad Amin Kerachian (n = 128) and Michael A Mont (n = 123). Clearly, the results of this Ronald S Weinstein study lay the theoretical and experimental foundation for the study of PCD in ONFH.

We selected the 20 most cocited coreferences to determine the research basis for PCD in ONFH. "Glucocorticoids in osteonecrosis of the femoral head: A new understanding of the mechanisms of action" published by Mohammad Amin Kerachian et al. published the most cited study in 2009. This study indicated that the inhibition of osteoblast and osteoclast precursors, increased apoptosis of osteoblasts and osteocytes, prolonged osteoclast longevity and endothelial cell apoptosis were both direct effects of glucocorticoid use. Thus far, glucocorticoids and apoptosis remain the focus of PCD in ONFH. Professor Robert S. Weinstein has published four of the 20 cited papers, Professor Michael A Mont three and Professor Mohammad Amin Kerachian two, which were the basis of PCD in ONFH.

In terms of the references with citation bursts, we found that studying the biological role and pathogenesis of glucocorticoid-induced osteonecrosis was the main research content of the strong citation burst references related to PCD in ONFH. In addition to citation bursts, keywords could help us quickly capture the distribution and evolution of hot spots in the field of PCD in ONFH. The study of ONFH involves its etiology, pathogenesis, diagnosis, treatment and prevention. In addition to keywords such as osteonecrosis, glucocorticoid, apoptosis and autophagy, Table 6 mainly includes the following keywords: angiogenesis, oxidative stress, exosomes, bone marrow mesenchymal stem cells, osteogenic differentiation and microRNA. Based on the keyword clustering analysis and trend topic analysis, we concluded that autophagy was most likely to be the current research hot spot for PCD in ONFH. Autophagy is activated upon starvation or stress, maintaining tissue function and homeostasis. The production of autophagy is dependent on lysosomal catabolism to degrade aged or damaged proteins and organelles into amino acids and fatty acids for energy production and recycling.

Of course, this study also had some drawbacks. The study analyzed the literature on ONFH and PCD research in the WoSCC database, and other databases were ignored. The article included only the literature in English, possibly overlooking high-quality literature in the field of ONFH and PCD in other languages. Therefore, it was possible to miss some relevant studies, which might bias the results; future analysis could include more databases and non-English-language papers. This study also had unique strengths. For instance, we were innovative in our systematic analysis of the research status of the field of PCD in ONFH through bibliometric analysis and provided speculations on the future development directions. These contributions could provide more guidance for scholars performing relevant research.

## Conclusions

In summary, this paper has important research value and application prospects in PCD and ONFH. The rapid increase in the number of publications suggested that the study of PCD in ONFH is increasingly valued by scholars worldwide. The countries involved with this research were mainly China, USA and Japan. China is currently in the lead in the field of PCD in ONFH. The quantity and quality of the literature in China ranked first in the world but showed a lack of extensive international cooperation, and the cooperation and communication between countries and institutions still needs to be strengthened. Furthermore, autophagy is a recent research hot spot, but with processes that represent the deepening of PCD in ONFH, such as pyroptosis, ferroptosis and cuproptosis, it is speculated that there will be additional room for research on PCD in ONFH in the future. Additionally, bibliometric and visual analysis need a substantial and comprehensive body of literature data to yield results, and researchers need to pay close attention to the development of PCD and ONFH in the future to track the latest developments in the field.

## Data Availability

Publicly available datasets were analyzed in this study. The Web of Science Core Collection database can be found at the following URLs: https://www.webofscience.com/wos/woscc/basic-search.

## Abbreviations

JCR: Journal Citation Reporting
ONFH: Osteonecrosis of the femoral head
PCD: Programmed cell death
WoSCC: Web of Science Core Collection
